# Protein–protein interactions in bacteria: a promising and challenging avenue towards the discovery of new antibiotics

**DOI:** 10.3762/bjoc.14.267

**Published:** 2018-11-21

**Authors:** Laura Carro

**Affiliations:** 1 School of Pharmacy, University of East Anglia, Norwich Research Park, Norwich NR4 7TJ, UK

**Keywords:** new antibiotics, protein–protein interactions, resistance

## Abstract

Antibiotics are potent pharmacological weapons against bacterial infections; however, the growing antibiotic resistance of microorganisms is compromising the efficacy of the currently available pharmacotherapies. Even though antimicrobial resistance is not a new problem, antibiotic development has failed to match the growth of resistant pathogens and hence, it is highly critical to discover new anti-infective drugs with novel mechanisms of action which will help reducing the burden of multidrug-resistant microorganisms. Protein–protein interactions (PPIs) are involved in a myriad of vital cellular processes and have become an attractive target to treat diseases. Therefore, targeting PPI networks in bacteria may offer a new and unconventional point of intervention to develop novel anti-infective drugs which can combat the ever-increasing rate of multidrug-resistant bacteria. This review describes the progress achieved towards the discovery of molecules that disrupt PPI systems in bacteria for which inhibitors have been identified and whose targets could represent an alternative lead discovery strategy to obtain new anti-infective molecules.

## Introduction

Bacterial infections are not only one of the most frequent diseases in humans and livestock, but also one of the top ten causes of death according to the World Health Organization [1]. The serendipitous discovery of penicillin and its introduction into the clinic in the first half of the 20th century gave rise to the “Golden Age” of antibiotics discovery and have unquestionably represented one of the most important achievements in medicine. Unfortunately, since their use is intrinsically linked to the appearance of resistance, threatening antibiotic-resistant bacteria levels are being observed, compromising the efficacy of nearly all available antibiotics to cure infectious diseases [2–4].

The rise in the percentage of antibiotic-insensitive strains, the steady decline in the number of new antibacterial drugs and the insufficient investment in antimicrobial research and development (R&D) by the major pharmaceutical companies have led to a global health crisis in which the prospect of a future without a safe and effective anti-infective compound is a very real and alarming possibility [5–6]. Consequently, new antibacterial drugs and treatment strategies are urgently needed to tackle the increasing multidrug-resistance in bacteria [7].

To further accelerate antibiotics development numerous approaches have been put in place. For example, the WHO recently published a list of global priority antibiotic-resistant bacteria to help prioritizing the research and development of new and effective antibiotic treatments [8]. In this list the pathogens were ranked in three priority levels according to the species and type of resistance:

1. Priority 1 – Critical:

- *Acinetobacter baumannii*, carbapenem-resistant
- *Pseudomonas aeruginosa*, carbapenem-resistant
- *Enterobacteriaceae*, carbapenem-resistant, 3rd generation cephalosporin-resistant

2. Priority 2 – High:

- *Enterococcus faecium*, vancomycin-resistant
- *Staphylococcus aureus*, methicillin-resistant, vancomycin intermediate and resistant
- *Helicobacter pylori*, clarithromycin-resistant
- *Campylobacter*, fluoroquinolone-resistant
- *Salmonella* spp., fluoroquinolone-resistant
- *Neisseria gonorrhoeae*, 3rd generation cephalosporinresistant, fluoroquinolone-resistant

3. Priority 3 – Medium:

- *Streptococcus pneumoniae*, penicillin-non-susceptible
- *Haemophilus influenzae*, ampicillin-resistant
- *Shigella* spp., fluoroquinolone-resistant

Given the prevalence of antibacterial drug-resistant pathogens, one viable and promising strategy to combat these multidrug-resistant bacteria is the development of antibiotic therapies with novel unconventional targets [9], such as protein–protein interactions [10–11].

This review covers the recent medicinal chemistry efforts towards the discovery of antibacterial molecules that disrupt protein–protein interactions (PPIs) by interacting directly to the protein–protein binding interface in both Gram-negative and Gram-positive microorganisms. In order to encourage prospective drug discovery endeavours in this field, this study focuses on four examples of bacterial PPIs for which inhibitors with promising activities have been reported. For each of the targets the structural features of the ligands, the discovery strategy, the characterization assay, the biological activity, and, if applicable, the SAR are discussed.

## Review

### Protein–protein interactions

Due to the fact that protein–protein interactions (PPIs) play a pivotal role in many cellular processes, they have increasingly become an attractive target over the past two decades [12–14].

**Figure 1:**
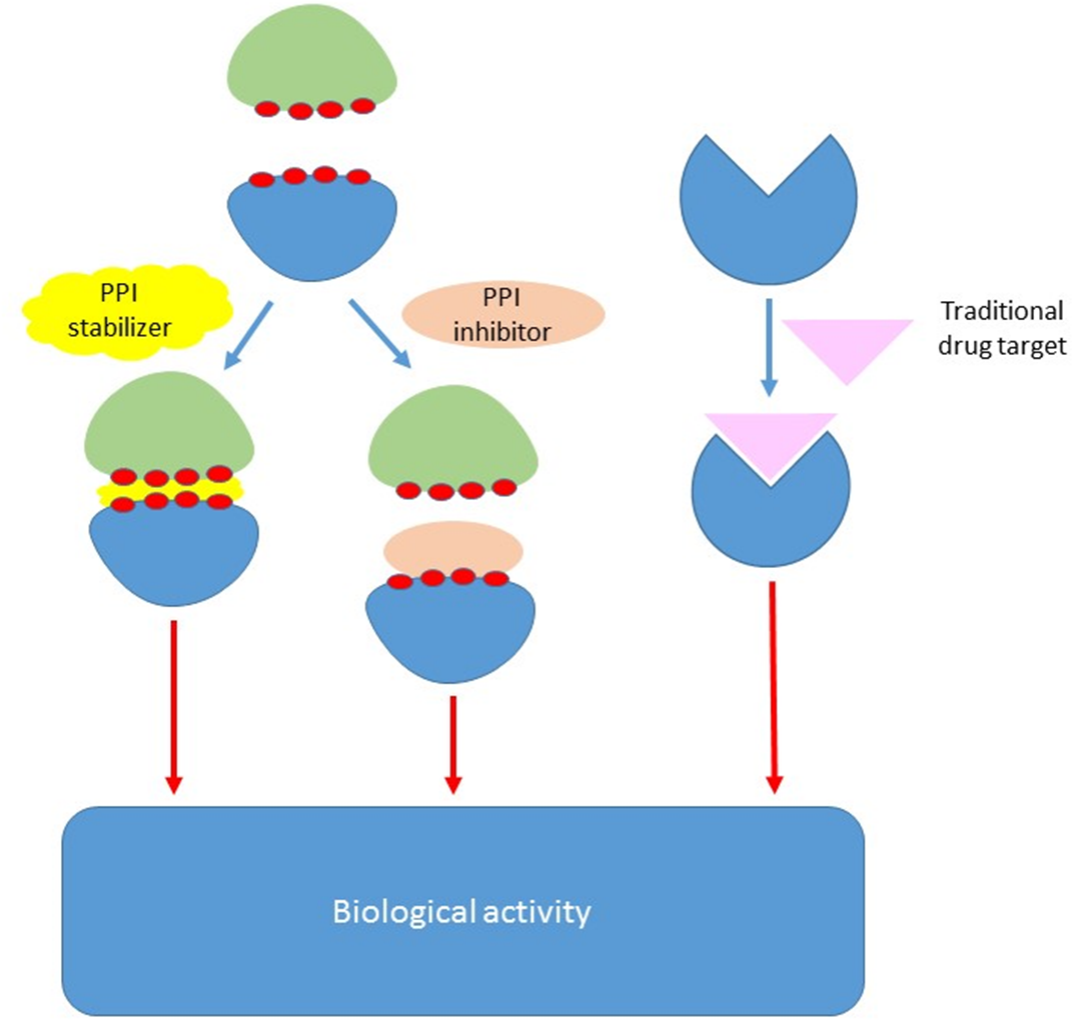
Illustration of a PPI modulator (stabilizer or inhibitor) vs a traditional drug target.

PPIs are challenging targets because of their flat, large and hydrophobic binding surface, in comparison with the well-defined binding sites of more classical targets such as GPCRs, enzymes or ion channels (Figure 1). Moreover, PPIs, unlike the previous examples, do not have a small natural ligand which can be used as a lead in a standard drug development programme [15–16]. Despite the binding surfaces being large, it is well known that not all the amino acid residues at the interface contribute equally to the binding, but in fact there are focal points (i.e., hot spots or hot segments) that contribute to the majority of the binding energy [17–18]. Targeting these “druggable” sites can therefore be used for the rational design of new therapeutic compounds that can disrupt those critical interactions. However, their identification requires detailed structure elucidation, which in the end makes the design of an effective PPI modulator both difficult and challenging [19–22]. PPI modulation can be achieved through two opposite but complementary approaches: stabilization or inhibition (Figure 1). Although so far the vast majority of protein–protein interaction modulators execute their activity through inhibition, stabilization of specific protein complexes could also be therapeutically beneficial [23–24].

Even though historically PPIs have been considered to be “undruggable”, recent remarkable medicinal chemistry efforts, mainly due to the development and implementation of more sophisticated screening methods and synthetic procedures, have led to the identification and clinical development of chemical entities that disrupt protein–protein interactions [15,25–26].

**Figure 2:**
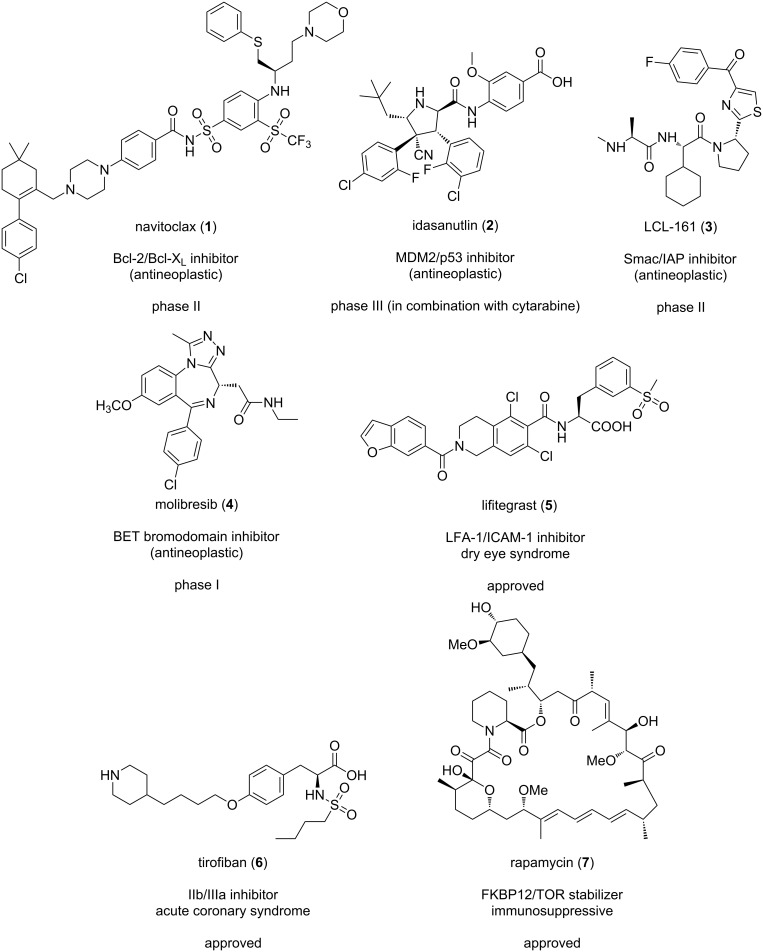
Examples of protein–protein interaction modulators in clinical trials or in clinical use.

A selection of a few PPI modulators that have recently been approved or reached clinical validation can be found in Figure 2. If we analyze their mechanism of action, nearly all of them are currently being investigated as oncological treatments. For example, navitoclax (**1**, Figure 2), a Bcl-2/Bcl-X_L_ inhibitor developed by Abbot Laboratories is currently in phase II for the treatment of several types of cancers [27]. Idasanutlin (**2**, Figure 2) from Hoffmann-La Roche, targets the extensively studied interaction between MDM2/p53 and is currently in phase III for the treatment of refractory acute myeloid leukemia in combination with cytarabine [28]. LCL-161 (**3**, Figure 2), an inhibitor of the interaction between Smac (second mitochondria-derived activator of caspases) and IAP (inhibitor of apoptosis proteins) developed by Novartis, has recently entered phase II for the treatment of leukaemia [29]. Another example is the inhibitor of the BET (bromodomain and extra terminal) molibresib (**4**, Figure 2), developed by GSK and currently in phase I for the treatment of several carcinomas [30]. It is also worth highlighting two PPI inhibitors that have recently been approved: lifitegrast (**5**, Figure 2) is an anti-inflammatory integrin antagonist that disrupts the LFA-1/ICAM-1 interaction used for the treatment of dry eye disease [31–32], and tirofiban (**6**, Figure 2), a platelet glycoprotein IIb/IIIa inhibitor indicated in acute coronary syndrome [33]. In addition to small molecules, natural products have been shown to be able to modulate protein–protein interactions and have validated PPI stabilization as a biological target. One of the most prominent examples of PPI-stabilizing natural products that are currently used in the treatments of human diseases is rapamycin (**7**, Figure 2), an immunosuppressant that inhibits the protein kinase TOR (target of rapamycin) [34]. This natural product, isolated from *Streptomyces hygroscopicus*, was one of the first protein–protein interaction stabilizers reported: it first binds to its receptor (i.e., FKBP12) with high affinity, after which the FKBP12-rapamycin complex will associate with TOR resulting in inhibition of the catalytic activity of the enzyme [23].

All these drug discovery successes have validated PPIs as a target and, in conjunction with the elucidation and reconstruction of protein–protein interaction networks in bacteria, have paved the way towards the development of novel and promising inhibitors of PPIs which may find application as anti-infective therapies.

### Protein–protein interactions in bacteria

Like in eukaryotes, protein–protein interactions are essential in prokaryotic cells in which they also have a central role. They coordinate many bacterial physiological processes such as regulation of gene expression, DNA replication, signal transduction, virulence, etc. and therefore represent potential fruitful targets for antibacterial drug discovery.

Recently, scientific efforts have helped towards the understanding and the deciphering of the protein-interaction networks (PINs) that forge the bacterial interactome [35]. However, despite the potential of these bacterial PPI maps, they have only been studied in detail in a few microorganisms including *Escherichia coli* (one of the best-studied model organisms in this field) [36–39], *Mycobacterium tuberculosis* [40], *Helicobacter pylori* [41], *Pseudomonas aeruginosa* [42], *Campylobacter jejuni* [43], *Treponema pallidum* [44], the cyanobacterium *Synechocystis* spp. [45], *Mesorhizobium loti* [46] and *Mycoplasma pneumoniae* [47]. Furthermore, partial PINs for *Bacillus subtilis* [48] and *Streptococcus pneumoniae* [49] have been reported recently, and many more are near completion [50].

These hundreds of thousands of known interactions, and those which are yet to be discovered, have been and will be essential for identifying potential points of intervention in clinically relevant pathogens that can serve as drug targets for antibacterial therapy. There are several reasons that support this argument: they are essential for not only the growth but the reproduction of the cells, they are conserved across many strains of pathogens and, most importantly, they are specific to the prokaryotic kingdom, meaning that either these interactions are non-existent or substantially different from their corresponding processes in eukaryotic cells [51].

Four examples of protein–protein interaction systems in bacteria for which inhibitors have been discovered and that could represent an alternative lead-discovery strategy to obtain new antimicrobial molecules are presented below.

### β-Sliding clamp

**Figure 3:**
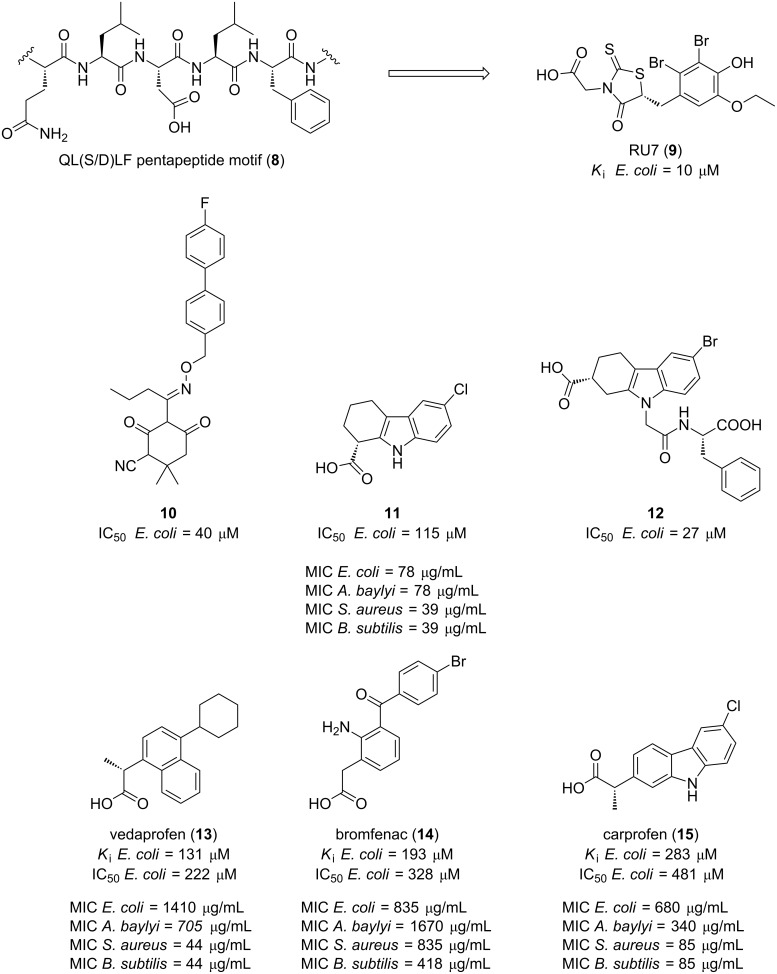
Small-molecule inhibitors of PPIs in the β-sliding clamp.

Sliding clamps are prokaryotic ring-shaped proteins that secure DNA polymerases to the DNA template and slide along the double helix, enabling enzyme activity at a specific region of the DNA and increasing the rapid and processive DNA synthesis [52–54]. The β-clamp interacts with many different proteins such as several polymerases (e.g. I, II, III, IV, V and DnaE) and other proteins involved in DNA replication including DNA ligase and the replication regulatory protein Hda, all of which contain the conserved binding pentapeptide motif QL(S/D)LF (**8**, Figure 3) [55–56].

There are several reasons that make the bacterial clamp a promising and attractive target for the development of new antibiotics: it is essential for DNA replication and repair, it is highly conserved across the different bacterial pathogens and, most importantly from a drug discovery perspective, its structure differs significantly from the eukaryotic equivalent clamp (PCNA, proliferating cell nuclear activity) [51,57].

**Figure 4:**
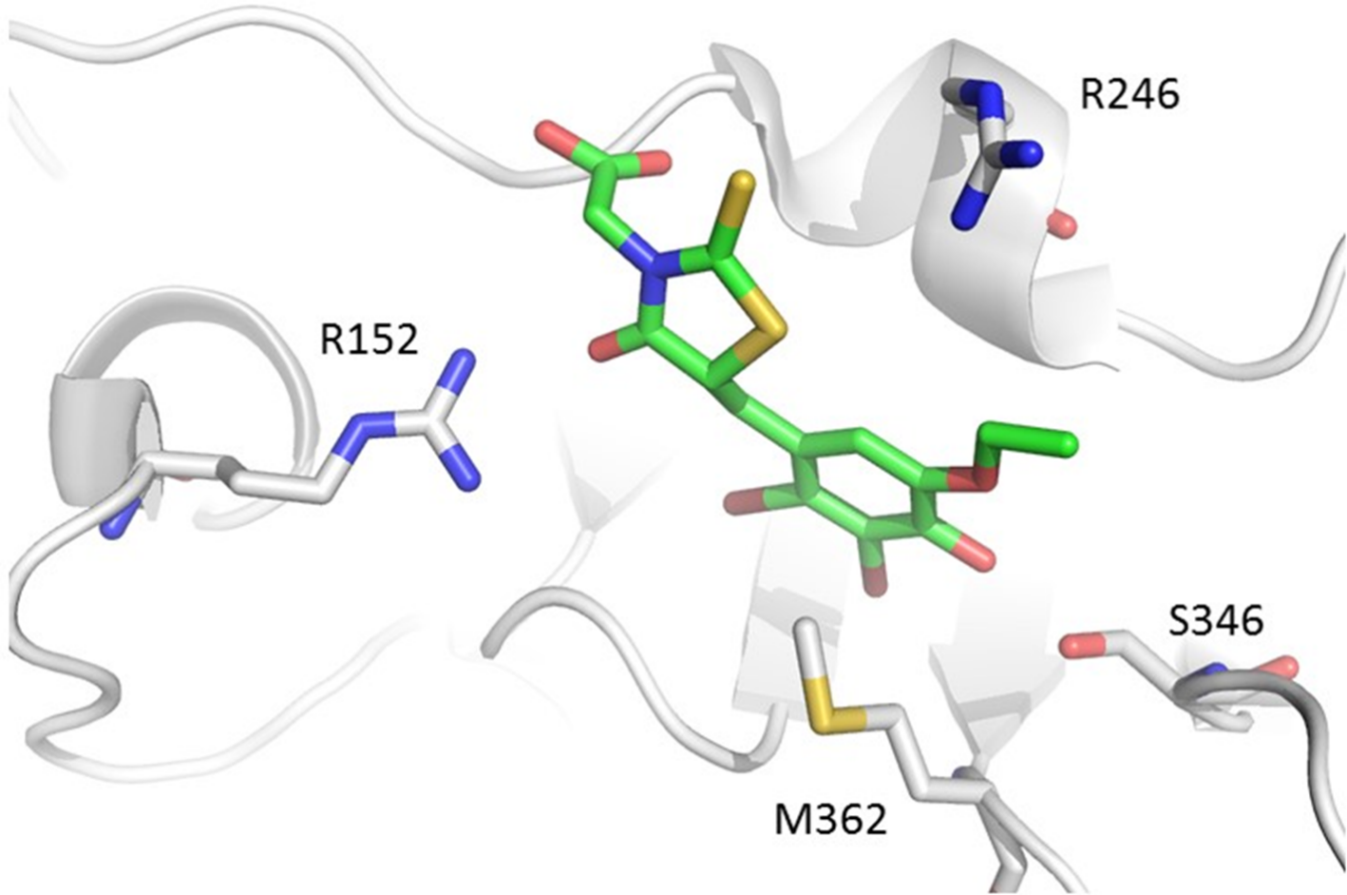
Crystal structure of the RU7 (**9**)-sliding clamp complex (PDB code 3D1G; adapted from Georgescu et al. [58]). Essential residues R152, M362, S346 and R246 are highlighted (sticks).

O’Donnell et al. first identified the structure of an inhibitor of the *E. coli* sliding clamp (ECSC) [58]. In the search for a molecule that binds to the peptide-binding pocket of the β-clamp, they carried out a fluorescence anisotropy titration screening of the Rockefeller University chemical library containing 30,600 polar organic compounds which led to the discovery of RU7 (**9**, Figure 3) with an inhibition constant of 10 μM. Pleasingly, it was also found that RU7 selectively disrupts the *E. coli* β-clamp without affecting its eukaryotic counterpart in *Saccharomyces cerevisiae*. In this same study, the co-crystal structure of RU7 with the sliding clamp revealed that the inhibitor occupies the deepest subsite (i.e., 1) of the two subsites that form the binding pocket (Figure 4) [58–59].

Utilizing a virtual screening of two different databases (i.e., the National Cancer Institute [60] and an in-house collection of 32,000 compounds), Wijfells et al. were able to identify a small-molecule mimic of the *des*-amino-Leu-Phe (dLF) component of the ECSC **10** (Figure 3), which displayed an IC_50_ in the low micromolar range (IC_50_ = 40 μM). X-ray crystallography studies revealed that this biphenyl oxime derivative **10** also occupies subsite I of the β-clamp [61].

In 2014, the Zenobia’s First Pass Screen fragment library containing more than 350 fragments was screened by X-ray crystallography leading to the identification of four fragment hits. In an attempt to improve their binding affinities, another library was searched for compounds displaying similarity to these initial hits. After a docking-based screening followed by a fluorescence polarization (FP) assay of the selected candidates, the substituted tetrahydrocarbazole **11** (Figure 3) was found to not only completely occupy *E. coli* SC subsite I with the highest affinity (IC_50_ = 115 μM) and to inhibit in vitro DNA replication, but also to have antibacterial activity against several Gram-positive and Gram-negative pathogens, namely *Bacillus subtilis*, *Staphylococcus aureus, Escherichia coli and Acinetobacter baylyi* with MICs ranging from 39 to 78 μM [62]. A year later, further SAR investigations from the same research group led to the identification of another tetrahydrocarbazole derivative **12** (Figure 3) which displayed a >4-fold increase in its affinity for *E. coli* SC [63].

Recent evidence suggests that non-steroidal anti-inflammatory drugs (NSAIDs) have antimicrobial activity. Oakley et al. studied the *E. coli* β-clamp binding affinity of commercially available NSAIDs with the help of a FP competition assay. Of the twenty compounds evaluated, five showed *K*_i_ values in the high micromolar range, but only vedaprofen, bromfenac and carprofen (**13**–**15**, Figure 3) displayed the strongest effects (*K*_i_ < 300 μM) [64]. Similarly to the preliminary study by Yin et al. [62] the antibacterial activity of the selected NSAIDs was determined on four clinically relevant species, two Gram-negative bacteria (*E. coli* and *A. baylyi*) and two Gram-positive (*S. aureus* and *B. subtilis*). In opposition to Yin’s studies, the latter species showed higher susceptibility than the Gram-negative bacteria with minimal inhibitory concentrations as low as 44 μg/mL in the case of vedaprofen (**13**). Again, and in agreement with previous studies, the compounds that most potently inhibited *E. coli* β-clamp binding also showed the highest level of antibacterial activity, supporting the correlation between inhibition of the sliding clamp and the antibacterial effects.

In addition to small molecules, peptides have also been investigated as disruptors of protein–protein interactions in the sliding clamp.

**Figure 5:**
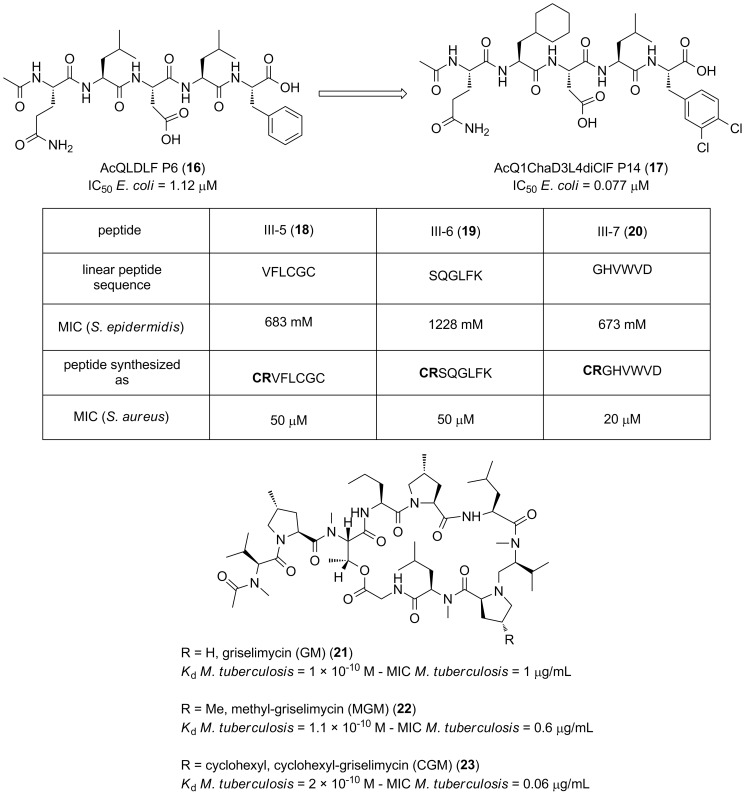
Peptidic inhibitors of PPIs in the sliding clamp.

A structure-based approach, using the natural pentapeptide QL(S/D)LF (**8**, Figure 3) as a template, led to the identification of the short peptide P6 (**16**, Figure 5) with enhanced affinity for the β-clamp (IC_50_ = 1.12 μM, measured by surface plasmon resonance). This acetylated peptide was used as a lead and further modified at the second position, where the leucine residue was replaced by a cyclohexyl-L-alanyl group (Cha), and also on the terminal phenylalanine benzyl ring, in which two chlorine atoms where incorporated in the benzene ring to achieve P14 (**17**, Figure 5) with a 15-fold increase of its binding affinity for the sliding clamp and reaching the 10^−8^ M range (IC_50_ = 0.077 μM) [65].

Recently, Løbner-Olesen and co-workers screened a library of peptides for their ability to disrupt PPIs in the β-sliding clamp of *Staphylococcus aureus*. In this elegant study, from a library of 900,000 cyclic peptides, which was intracellularly generated using the split-intein circular ligation of peptides and proteins (SICLOPPS) technology [66], three hits, peptides III-5, III-6 and III-7 (**18**–**20**, Figure 5), were identified to be able to disrupt the DnaN–DnaN (β-clamp–β-clamp) interaction. Interestingly, III-5 (**18**) and III-6 (**19**) were able to inhibit the growth of *Staphylococcus aureus* with MIC values of approximately 50 μg/mL while their linear counterparts displayed no activity [67].

Historically, natural products have been one of the most fruitful sources to obtain antibacterial lead compounds [5,68–69]. Griselimycin, a cyclic depsidecapeptide isolated from *Streptomyces* sp., was discovered fifty years ago, nonetheless, due to its poor pharmacokinetic properties and the availability of other drugs such as rifampicin, the optimization programme was abandoned [70]. Recently, the interest in neglected antibiotics with anti-tuberculosis potential resurged and led to further optimization and development studies around the griselimycin structure (**21**, Figure 5) [71]. Müller et al. discovered that griselimycin and its metabolically more stable analogues (methyl-griselimycin, MGM, **22** and cyclohexyl-griselimycin, CGM, **23**) were active against *M. tuberculosis* in the low micromolar range with MICs of 1, 0.6 and 0.06 μg/mL, respectively. To characterize the target protein of griselimycins, surface plasmon resonance (SPR) was used, analysis that demonstrated that they bound with high affinity to the sliding clamps of *M. tuberculosis* (*K*_d_ ranging from 1.0 × 10^−10^ M to 2.0 × 10^−10^ M). Encouragingly, no binding was detected between griselimycins and the human sliding clamp, and, hence, exhibiting an excellent selectivity profile. Crystal structures revealed that GM (**21**) and CGM (**23**) bind to a hydrophobic pocket between domains II and III, the protein–protein interaction site responsible for the recruitment of DNA enzymes by the sliding clamp, and therefore promisingly validating the sliding clamp as a feasible antibacterial target.

### Single-stranded DNA-binding protein (SSB)

SSB is a class of proteins that coordinates fundamental cell processes including DNA replication, recombination and repair, and is, consequently, vital for cell survival. In addition, it also mediates the binding to more than fourteen genome maintenance proteins of the SSB interactome [72]. This latter activity enables SSB to act as a conserved hub of proteins which recruits their binding partners (e.g., exonuclease I, the DNA primase DnaG and the DNA helicase PriA) to their site of action [73].

Similarly to the aforementioned sliding clamp, the structural arrangement of most bacterial SSBs differs significantly to its homolog in eukaryotic cells (replication protein A, RPA) [74]. This dissimilarity could be beneficial from a drug discovery perspective because it would enable selective targeting of the bacterial interactome without impacting the eukaryotic cell processes.

**Figure 6:**
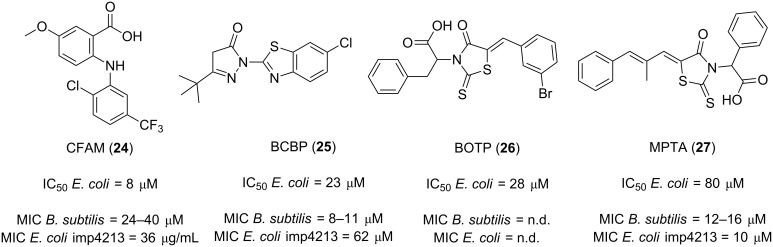
SSB protein–protein interaction inhibitors identified by HTS.

Keck and co-workers identified four small molecules that disrupt the *Escherichia coli* SSB interaction with one of its well-studied binding partners, exonuclease I (ExoI) [75]. The screening by means of a high-throughput fluorescence polarization assay of 50,400 compounds from the Chemical Diversity, Maybridge and Chembridge chemical libraries, and subsequent dose-dependent evaluation of the disruption of the SSB/ExoI complex, led to the discovery of four inhibitors, CFAM, BCBP, BOTP and MPTA (**24**–**27**, Figure 6), with IC_50_ values ranging from 8–80 μM. Afterwards, the scientists were able to successfully obtain the crystal structures of the complexes of ExoI with both CFAM (**24**) and BCBP (**25**). From the crystallography studies it was revealed that, even though both compounds bind to the B site of ExoI, only CFAM (**24**) is able to interact with the crucial residues in the basic ridge which are known to be essential for the in vitro complex formation of ExoI with SSB [73], a finding that supports the low IC_50_ value exhibited by this molecule. Remarkably, these four compounds were also able to disrupt the complex formation of SSB with some other binding partners such as the DNA helicases RecQ and PriA but, unfortunately, less potently.

In an attempt to prove the hypothesis that direct targeting of PPIs, particularly SSB, could be an effective strategy for the development of novel broad-spectrum antibacterial agents, the colony formation evaluation of three of the previously mentioned hits (namely **24**, **25** and **27**) was assessed against a panel of bacterial strains that included Gram-positive microorganisms *Listeria monocytogenes*, *Staphylococcus aureus*, *Mycobacterium smegmatis*, *Mycobacterium tuberculosis*, *Mycobacterium avium paratuberculosis* and *Mycobacterium bovis*, and Gram-negative pathogens *Escherichia coli* (wt and imp4213), *Bacillus subtilis*, *Neisseria gonorrhoeae*, *Neisseria meningitidis* and *Neisseria lactamica* [76]. As a result, the compounds were able to successfully inhibit the growth of some of the bacteria tested. The authors also determined the growth suppressive effects on model Gram-positive (*B. subtilis*) and Gram-negative (*E. coli* imp4213) bacteria. The MIC values against *B. subtilis* were found to be 40, 11, 16 μM for CFAM, BCBP and MPTA, respectively, while the MIC values against the membrane-compromised *E. coli* were found to be 36, 62 and 10 μM, respectively.

High-throughput screening initiatives have gained popularity in the past two decades and have become the prevailing approach to identify leads in medicinal chemistry research [77–78]. However, due to the intrinsic features of PPIs, these are not amenable to the HTS approaches used to identify small molecules which will typically target enzymes (e.g., kinases and proteases) or extracellular receptors [79].

Recently, a HTS strategy to identify inhibitors of the *Klebsiella pneumonia* SSB PPI was reported. Starting from a library of more than 72,000 compounds from Life Chemicals Inc. (Munich, Germany), nine selective inhibitors of the SSB/PriA interaction with IC_50_ values of <40 μM were identified by orthogonal fluorescence polarization assays. Of these nine initial hits, two were observed to interact with PriA by thermal stabilisation in a differential scanning fluorimetry (DSF) assay [80]. Regrettably, no data was presented on the chemical structures of these compounds or the antibacterial activity. Nevertheless, this study reinforces the usefulness of integrating biophysical techniques with HTS approaches in order to detect and investigate SSB protein–protein interactions in bacterial systems.

**Figure 7:**
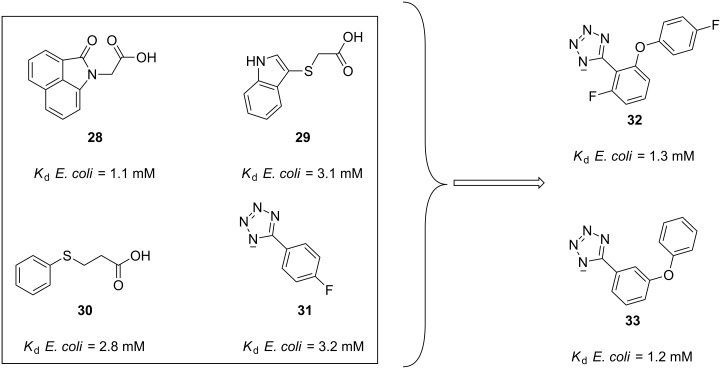
SSB protein–protein interaction inhibitors identified by FBDD.

In the same year, Oakley et al. implemented a fragment-based drug discovery (FBDD) approach in order to identify disruptors to the interaction of SSB with another of its partners, the DNA primase DnaG [81]. In this study, a SPR competition assay and a saturation-transfer difference NMR (STD-NMR) led to the identification of thirty fragments. Subsequent 2D-^15^N–^1^H HSQC NMR returned four fragment hits **28**–**31** (Figure 7), with binding affinities, determined by NMR titration, in the low millimolar range.

Of all of the fragments, tetrazole **31** was chosen for further optimization due to its physicochemical properties and its potential for fragment growth. After virtual screening and binding studies by STD-NMR studies, the authors were able to find tetrazole **32** (Figure 7) which had a three-fold improved affinity (*K*_d_ = 1.3 mM) compared to the initial hit **31**. Later, the ZINC database [82] was searched for structurally similar compounds leading to the identification of the *meta*-substituted tetrazole **33** (Figure 7), which was found to have a similar dissociation constant. Moreover, in order to predict the orientation of the fragments in the binding site, molecular docking of **33** to DnaG was predicted.

Finally, the fragments were also assessed against other SSB partner proteins by means of STD-NMR. Although the affinity values were not determined, all of them were satisfactorily found to bind to *E. coli* PriA, *E. coli* RNAse HI and the χ subunit of *E. coli* and *A. Baumannii* DNA polymerase III, and thus represent promising leads in the search for PPI inhibitors in bacteria.

### Filamenting temperature-sensitive protein Z (FtsZ)

FtsZ is a prokaryotic tubulin-like protein which plays a crucial role in cell division in both Gram-positive and Gram-negative bacteria [83]. This protein polymerizes into a ring structure (the Z-ring) early at the division site and operates as a focal point for the assembly of the other division proteins [84].

There are several advantages of cell-division proteins as an antibacterial target: first, their intrinsic characteristics and essentiality for the multiplication and viability of bacteria, and second, they are highly conserved in many bacterial species [85].

Various studies have shown that Z interaction protein A (ZipA) is one of the essential components that stabilize the Z-ring formation and that it binds to FtsZ with high affinity. The interaction between FtsZ and ZipA is essential for cell division in *E. coli* and other Gram-negative bacteria, and therefore, it has been suggested that disruption of the ZipA/FtsZ interaction can be exploited to develop potential antibacterial molecules [83,86].

**Figure 8:**
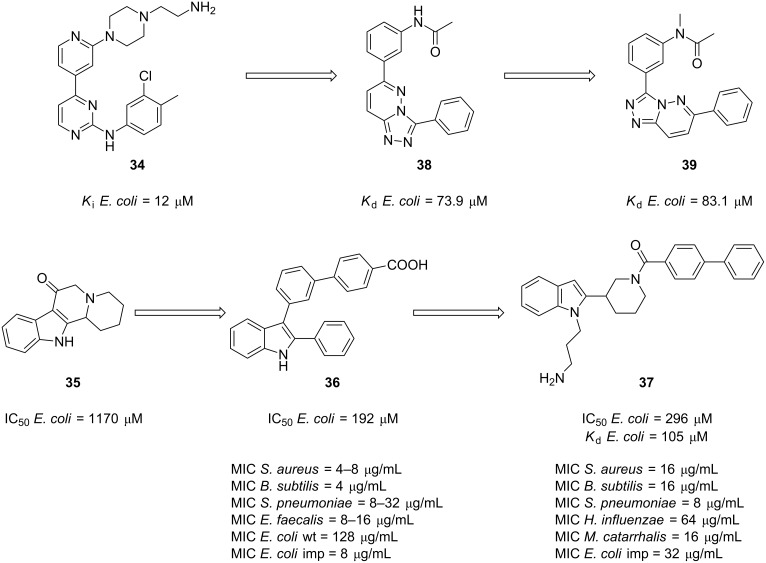
Examples of molecules that disrupt the ZipA/FtsZ interaction.

The development of a small-molecule antibiotic that targeted the ZipA/FtsZ protein–protein interaction was first investigated by scientists at Wyeth Research. In this study, a fluorescence polarization-based high-throughput screening of 250,000 corporate compounds led to the identification of pyridylpyrimidine **34** (Figure 8), which was shown to be the most potent with a *K*_i_ of 12 μM as measured by a FP competition assay [87]. Kenny et al. succeeded in obtaining a crystal structure of **34** bound to ZipA which provided detailed knowledge of the basis of the binding mode. Interestingly, it was found that, although **34** occupies only half of the ZipA/FtsZ binding pocket, the phenyl, the pyridine and the pyrimidine rings make critical hydrophobic interactions with residues in the shallow groove of the pocket [87].

Nearly simultaneously, the same group reported the SAR studies of a family of biphenylindole derivatives as inhibitors of the same PPI. A structure-based design built on the indoloquinolizinone **35** (Figure 8), afforded the chimeric molecule indole **36** (Figure 8) as an inhibitor of the ZipA/FtsZ interaction with an improved, but still modest, IC_50_ of 192 μM. The antibacterial activity of all the target compounds was also evaluated against a panel of microorganisms which included Gram-positive pathogens *Staphylococcus aureus*, *Streptococcus pneumoniae*, *Bacillus subtilis*, *Enterococcus faecalis* and the Gram-negative bacterium *Escherichia coli* (wt and an outer membrane permeable strain). Gratifyingly, the antibacterial screening showed that improved IC_50_ values correlated with improved minimal inhibitory concentrations and that Gram-positive microorganisms are more susceptible, likely due to the inability of molecules to permeate the outer membrane of Gram-negative pathogens [88].

In a follow-up study, a combinatorial synthesis approach was utilized to generate a library of small molecules whose inhibition properties were measured by a fluorescence polarization competition assay [89]. Derivatization of the indole nitrogen atom of lead compound **36** (Figure 8) returned the interesting indole analogue **37** (Figure 8). The authors then confirmed that the compounds were binding to the *E. coli* Zip A in the FtsZ binding site by means of 2D-HSQC NMR experiments and later submitted the selected compounds to cell division inhibition assays and MIC determination against a broad panel of bacterial strains which included *S. aureus*, *B. subtilis*, *S. pneumoniae*, *H. influenzae*, *M. catarrhalis*, and *E. coli* (imp). Unfortunately, the most active compound, indole **37**, exhibited an IC_50_ = 296 μM and a dissociation constant of 105 μM which undoubtedly make it a too weak inhibitor. Excitingly, the results are consistent with the inhibition of the ZipA/FtsZ interaction measured by FP and therefore indole **37** exhibited the best profile of in vitro cell growth inhibition with MICs ranging from 8–64 μg/mL.

Computational studies were also undertaken in an attempt to identify new inhibitors of the interaction between ZipA and FtsZ [90]. Utilizing the structure of the pyridylpyrimidine **34** (Figure 8) as a template, Rush et al. applied a shape-comparison program (rapid overlay of chemical structures, ROCS). This study led to the identification of three lead-like scaffolds among which compound **38** (Figure 8) was the most active with a *K*_d_ of 73.9 μM. In spite of the fact that this molecule was less active than the original lead, the authors argued that it had less development issues. Finally, in order to confirm the binding mode of these new structures, the crystal structure of **39** (*K*_d_ = 83.1 μM) in complex with ZipA was solved. Excitingly, the X-ray analysis revealed that ROCS very accurately predicted the binding mode and therefore validated its use as an alternative approach to identify new promising leads as inhibitors of this protein–protein interaction.

Despite the reported advances, these compounds were not found to be therapeutically relevant inhibitors of the ZipA/FtsZ protein–protein interaction, nonetheless considering the challenges involved in targeting PPIs, it is significant that the authors demonstrated by NMR that compounds from this library bind to ZipA at the FtsZ binding site and that small molecule disruptors of the ZipA/FtsZ interaction could inhibit cell division in both Gram-positive and Gram-negative microorganisms, findings that could be of value in the development of optimized antagonists for potential use as antibacterials.

### N-Utilization substances (Nus) B and E

Another point of therapeutic intervention to develop new anti-infectives that target cell viability is bacterial transcription, a process that is executed by the enzyme RNA polymerase (RNAP) and regulated by several transcription factors.

Similarly to the previously described targets, bacterial transcription represents a promising antibacterial drug target for several reasons: it is essential to cell viability, RNAP and its transcription factors are considerably conserved across many important bacterial strains and both of them differ from their eukaryotic homologs [91–92].

The transcription factors NusB and NusE and their interaction is vital for the efficient transcription in all bacteria [93] and have recently attracted interest as a potential target for a new antibiotic class.

**Figure 9:**
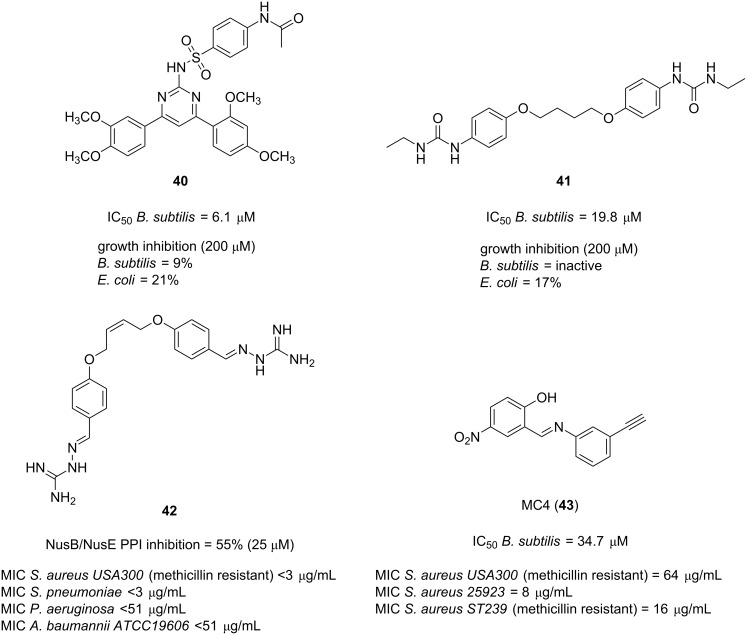
Inhibitors of the NusB/NusE interaction.

In 2017, McCluskey and co-workers carried out the virtual screening of 56,000 compounds from the mini-Maybridge library which led to the identification of five synthetically accessible hits [94]. In order to validate these in silico screened hits, their ability to inhibit the *Bacillus subtilis* NusB/NusE PPI was examined. Gratifyingly, compounds **40** and **41** (Figure 9) exhibited an inhibition of the NusB/NusE interaction at 25 μM higher than 50% and IC_50_ values in the low micromolar range (6.1 and 19.8 μM, respectively). A subsequent antibacterial screening showed that the lead pyrimidine **40** was also a moderate inhibitor of the growth of the Gram-positive pathogen *B. subtilis* and the Gram-negative microorganism *E. coli*.

The same research group developed further SAR studies using compound **41** (Figure 9) as lead of interest [95]. To this end, four focused compound libraries based on this bis-ether were developed leading to the identification of the rigidified *cis*-butene aminoguanidine analogue **42** (Figure 9) as both a good inhibitor of the NusB/NusE PPI (>50% at 25 μM) and a potent antibacterial against not only Gram-positive pathogens such as methicillin-resistant *S. aureus* and *S. pneumoniae* but also Gram-negative bacteria strains such as *P. aeruginosa* and *A. baumannii* (MIC ≤ 3 μg/mL and ≤51 μg/mL, respectively) [95]. In spite of the efficiency of the bis-aminoguanidine derivative **42**, unfortunately, its toxicity was an issue. Nonetheless, this study represents a step forward towards the potential development of next-generation anti-transcription antibiotics and validates the correlation between the NusB/NusE PPI and the in vitro antibiotic effect.

The first specific inhibitor against *S. aureus*, including MRSA, was reported very recently by Ma et al. [96]. After an in silico screening of a combination of the previously mentioned mini-Maybridge library and the Enamine antibacterial library, seven hits were identified. Among all of them, the nitrophenol derivative MC4 (**43**, Figure 9) was able to inhibit NusB/NusE binding with an IC_50_ of 34.7 μM. Its antimicrobial properties were also evaluated against a panel of clinically relevant microorganisms such as *Enterococcus faecalis*, *Klebsiella pneumonia*, *Acinetobacter baumannii*, *Pseudomonas aeruginosa*, *Enterobacter cloacae*, *Escherichia coli*, *Proteus vulgaris* and *Staphylococcus aureus*. However, in spite of the fact that NusB and NusE are highly conserved in bacteria, the compound exhibited preferred antibacterial activity against *S. aureus* strains (including MRSA) with minimum inhibitory values as low as 8 μg/mL. Pleasingly, unlike the aminoguanidine **42**, compound **43** did not display toxicity to mammalian cells.

Even though the NusB/NusE interaction is still in its infancy, and further investigations are needed to both elucidate off-target effects and apparent preferential inhibitory activity against Gram-positive pathogens, the identification of promising small molecules against these PPI (or other interactions involved in bacterial transcription yet to be discovered and validated) may have something to offer in the ongoing research towards the development of new anti-infectives with novel mechanisms of action.

## Conclusion

Pathogenic bacteria are a leading cause of human mortality, particularly in Third World countries. Due to the fact that new resistant microorganisms continue to emerge, combating these infections has become a global challenge for which the discovery and development of new antibacterial drugs is of critical importance.

Although progress has been made in order to address and overcome drug resistance, there is an urgent need to develop new antibacterial drug leads that operate through a novel mechanism of action.

On the other hand, the past two decades have observed the emergence of protein–protein interactions as a drug design target. During this time, many important studies have been conducted towards the identification and characterization of protein–protein interactions which have successfully resulted in several modulators reaching the clinical stage (Figure 1). These achievements would not have been possible without the utilization of appropriate design and screening approaches to determine the interactions at a molecular level and hence the development of PPIs as tractable targets.

To this end, interrogating interaction systems in prokaryotic cells that are critical for bacterial survival have recently become an attractive target which may offer therapeutically promising perspectives for infectious diseases.

In this review, the most significant compounds which have been found to disrupt protein–protein interactions in bacteria have been highlighted. These chemical scaffolds target different bacterial processes such as replication (SSB and sliding clamp), division (FtsZ) and transcription (NusB/NusE interaction). It is hoped that the knowledge acquired in both discovering and developing these inhibitors will lay the foundation for future antibacterial drug development pipelines. Given that the protein interactions systems described are conserved in prokaryotes but not present in eukaryotes, it may be feasible to develop inhibitors that selectively target the microorganism systems, and therefore avoiding mechanism-related side effects. However, these large sets of interactions remain poorly characterized and targeting them is a challenge.

Thus far, extensive in silico and high throughput screening campaigns of libraries of compounds, combinatorial synthesis and structure-based design approaches, biophysical screening techniques (i.e., fluorescence polarization, surface plasmon resonance and differential scanning fluorimetry) in combination with structural elucidation by combination of NMR and X-ray crystallography have played a pivotal role in identifying and deciphering the crucial target–inhibitor interactions with the aim of treating disease.

Although this review covers the description of PPI in bacteria, there are other strategies that are being explored towards the discovery of new antibiotics with novel mechanisms of action such as for example inhibitors of host–pathogen interactions and of the type 3 secretion system (T3SS) in Gram-negative bacteria, nonetheless, the specific targets remain unknown or ambiguous [97–98].

The challenge for new drug discovery efforts in the field of PPIs, which is still in the early stage, is to learn their real potential in combating infectious diseases. It is expected that enhanced understanding of the biology of bacteria and the nature of the PPI interfaces, in combination with medicinal chemistry efforts, may result in an opportunity to obtain antibacterial molecules whose mechanisms do not overlap with those of existing anti-infective drugs and consequently reduce the burden of multidrug-resistant pathogens.
